# Dysfunctional metacognition and drive for thinness in typical and atypical anorexia nervosa

**DOI:** 10.1186/s40337-015-0060-4

**Published:** 2015-07-04

**Authors:** Emily Davenport, Nola Rushford, Siew Soon, Cressida McDermott

**Affiliations:** Melbourne School of Psychological Sciences, Faculty of Medicine, Dentistry and Health Sciences, The University of Melbourne, Redmond Barry Building, Victoria, 3010 Australia; The Eating Disorders Unit, Royal Melbourne Hospital, Melbourne, Australia

**Keywords:** Anorexia nervosa, Atypical anorexia nervosa, Metacognition, Drive for thinness

## Abstract

**Background:**

Anorexia nervosa is complex and difficult to treat. In cognitive therapies the focus has been on cognitive content rather than process. Process-oriented therapies may modify the higher level cognitive processes of metacognition, reported as dysfunctional in adult anorexia nervosa. Their association with clinical features of anorexia nervosa, however, is unclear. With reclassification of anorexia nervosa by DSM-5 into typical and atypical groups, comparability of metacognition and drive for thinness across groups and relationships within groups is also unclear. Main objectives were to determine whether metacognitive factors differ across typical and atypical anorexia nervosa and a non-clinical community sample, and to explore a process model by determining whether drive for thinness is concurrently predicted by metacognitive factors.

**Methods:**

Women receiving treatment for anorexia nervosa (*n* = 119) and non-clinical community participants (*n* = 100), aged between 18 and 46 years, completed the Eating Disorders Inventory (3^rd^ Edition) and Metacognitions Questionnaire (Brief Version). Body Mass Index (BMI) of 18.5 kg/m^2^ differentiated between typical (*n* = 75) and atypical (*n* = 44) anorexia nervosa. Multivariate analyses of variance and regression analyses were conducted.

**Results:**

Metacognitive profiles were similar in both typical and atypical anorexia nervosa and confirmed as more dysfunctional than in the non-clinical group. Drive for thinness was concurrently predicted in the typical patients by the metacognitive factors, *positive beliefs about worry*, and *need to control thoughts;* in the atypical patients by *negative beliefs about worry* and, inversely, by *cognitive self-consciousness,* and in the non-clinical group by *cognitive self-consciousness.*

**Conclusions:**

Despite having a healthier weight, the atypical group was as severely affected by dysfunctional metacognitions and drive for thinness as the typical group. Because metacognition concurrently predicted drive for thinness in both groups, a role for process-oriented therapy in adults is suggested. Implications are discussed.

## Background

Medical and psychosocial complications of anorexia nervosa (AN) are severe, limiting development in all life domains. Compared with other psychiatric disorders, long-term outcome for anorexia nervosa is generally poor, with the highest mortality rate of any psychiatric disorder [1]. At twelve-year follow-up, 9.4 % of sufferers were deceased, with a very high standardised mortality rate of between 4.9-9.6 % [2]. Furthermore, a predictor of chronicity is chronicity itself [2]; the longer anorexia nervosa persists, the less effective therapy will be. No treatment has been consistently effective, undermined by delayed treatment onset [3, 4], premature treatment dropout of approximately 40 % [5], and failure to sustain change [6]. Consequently, the evidence base for AN treatment is poor [4, 7, 8], with treatment decision-making instead supported by clinical guidelines [9, 10].

Even for psychological interventions that have shown promise, such as enhanced cognitive behavioural therapy, failure of outpatients to complete treatment remains high [11, 12], attributed in part to a limited understanding of the specific cognitive and emotional processes that control, correct, appraise and regulate thinking in anorexia nervosa [13–15]. This has led to the development of intervention models [16, 17] focusing on cognitive *processes,* or metacognition, rather than cognitive *content* as in the cognitive behavioural model.

Metacognition is defined as ‘knowledge about one’s own thoughts and the factors that influence one’s thinking’ [18]. Wells & Matthews’ Self-Regulatory Executive Function (S-REF) model which links metacognition to psychopathology [19], proposes that distress in psychological disorders is generated and maintained by cognitive biases that arise from dysfunctional metacognition. The distorted metacognitions contribute to a maladaptive style of thinking termed the cognitive attentional syndrome (CAS) which is characterised by repetitive and difficult-to-control worry and rumination, threat-monitoring, self-focused attention, processing of negative self-beliefs and unhelpful coping behaviours, while difficulties in set-shifting prevent the acquisition of more adaptive knowledge [16, 19, 20]. For anorexia nervosa specifically, the metacognitive dysfunctions are thought to manifest in ruminations over distorted cognitions related to food, weight and shape, preventing individuals from constructive cognitive processing such as problem solving [21, 22]. Although metacognition is seen as trans-diagnostic [20, 23] across a range of mental disorders, metacognitions have been examined infrequently in eating disorders [19, 22].

Dysfunctional metacognition is operationalised through the Metacognitions Questionnaire (MCQ) [24], which assesses metacognitive beliefs, judgments, and monitoring tendencies in five replicable factors. These factors are *positive beliefs about worry, negative beliefs about worry*, *cognitive confidence*, *beliefs about the need to control thoughts*, and *cognitive self-consciousness*. In patients with anorexia nervosa, each of the metacognitive factors, except for *positive beliefs about worry*, has been reported as significantly more dysfunctional than in controls [25–27]. In a qualitative study of eating disorder sufferers, positive and negative metacognitions contributed to the selection of unhelpful coping strategies, perseverative thinking and attentional bias on food, body image and weight [26], cohering with the CAS [20]. A recent review of trials of the associated cognitive remediation therapy (CRT) [20] suggested that while there was evidence for individual trial effectiveness, particularly in conjunction with other therapies, there was much that has not yet been explored, including its applicability to trans-diagnostic treatment and metacognition.

To establish the therapeutic salience of dysfunctional metacognitions to anorexia nervosa, their links to core pathology should be supported. The drive to be thinner is a key indicator of the intensity of core symptoms of anorexia nervosa and is central to its aetiology and maintenance [28]. The Drive for Thinness (DFT) subscale of the Eating Disorders Inventory-3 (EDI-3) [29] denotes perceptions, attitudes, and behaviours associated with an intense desire for thinness, such as an excessive preoccupation with weight, dieting, and avoiding weight gain [30, 31], a pattern that is consistent with the CAS cognitive style and thus likely to have strong metacognitive influences. *Positive beliefs about worry* have the potential to influence DFT through the beliefs that worrying about weight gain helps to maintain dietary restriction [25], and that worrying and threat-monitoring are advantageous [32] in order to problem-solve [33], self-motivate [34], assert self-control [25] and distract [23]. *Negative beliefs about worry* refer to beliefs that worry-related thought processes are uncontrollable and physically and mentally dangerous, often prompting attempted suppression of thoughts [22, 23] and an increase in the frequency and salience of thoughts related to drive for thinness [35, 36]. The *need to control thoughts* generates feelings of responsibility for controlling thoughts to prevent negative outcomes which, already low in anorexia nervosa [37, 38], may be loss of control over weight-related strategies [38]. Low c*ognitive confidence,* particularly in memory and attention, is related to repetitive checking [39] and an increased focus on food and weight reduction [25]. *Cognitive self-consciousness* can also narrow the focus onto anorexia-related cognitions through heightened awareness of cognitive processes, with their rigid monitoring and catastrophic appraisal of mundane events [40, 41] increasing salience of intrusive and distressing thoughts [42]. The present study investigates associations between the five MCQ metacognitive factors and drive for thinness in adults.

Recent changes to the diagnosis of eating disorders in the Diagnostic and Statistical Manual of Mental Disorders, Fifth edition (DSM-5) [43] led to a further step in the present study, the inclusion of the newly-defined group, anorexia nervosa of higher weight, or atypical anorexia nervosa (AN-at). It includes individuals with the clinical features of anorexia nervosa who do not meet the more stringent DSM-TR weight criterion for AN [44]. Little has been reported on the clinical characteristics of AN-at. Comparing typical anorexia nervosa (AN-t) and AN-at on the study variables, duration of the disorder and weight histories may help to clarify clinical distinctions between the groups. The results may indicate whether the normal-weight AN-at patients are recovering or in partial remission from AN, or are driven to lose weight at least as zealously as the AN-t group with consequences sufficiently severe to warrant intensive treatment. The results could help inform therapeutic decision-making. A normal-weight, community-based non-clinical (CNC) group was included, with which to compare the clinical groups, given previously reported relationships between MCQ factors and disordered eating in non-clinical samples [45].

A major aim of the study was to explore differences between typical and atypical anorexia nervosa in adults by comparing, firstly, drive for thinness and metacognition and, secondly, weight histories and duration of the disorder. Differences between the non-clinical and AN groups were also explored. The concurrent prediction of drive for thinness by the metacognitive factors would facilitate further group comparisons and generate material relevant to process-oriented therapies [18].

Hypothesis H1: DFT is significantly more extreme in AN-at compared with AN-at. Hypothesis 2: the five MCQ factors (*cognitive confidence, positive* and *negative beliefs about worry, cognitive self-consciousness* and *need to control thoughts)* are significantly more extreme in AN-t than in AN-at. Hypothesis H3: DFT and the MCQ factors in each of AN-t and AN-at groups are significantly greater than in CNC. Exploratory hypothesis H4: Duration of AN and weight histories in AN-t and AN-at are significantly different. Hypothesis H5: DFT is concurrently predicted by MCQ factors, in each of AN-t, AN-at and CNC.

## Methods

### Classification of the anorexia nervosa groups

Criteria for classification of AN-t and AN-at based on weight are undefined or unclear [46, 47]. According to the newly-specified severity groupings of AN [46], individuals with BMI ≥ 17 kg/m^2^ have mild AN, while those with AN-at can be just as medically compromised after rapid weight loss even if their weight is still in, or above, the normal range. For this study, the criterion value for separation into the two AN groups was chosen as BMI = 18.5 kg/m^2^ [48], with AN-t having BMI < 18.5 kg/m^2^ and AN-at having BMI ≥ 18.5 kg/m^2^.

### Participants

Participants were women who voluntarily participated according to the ethical requirements of the participating university and two hospitals, one public and the other private. General exclusion criteria included male gender and insufficient English to give informed consent and complete questionnaires.

The patient sample consisted of 119 women who met criteria for AN-t (*n* = 75) or AN-at (*n* = 44), 60 % of whom were in-patients, 24 % day-patients, and 16 % out-patients, consecutively recruited from the eating disorders units of both hospitals. There were no significant differences between them on the study measures (*p* > .05). All had undergone a diagnostic interview with a psychiatrist or psychiatric registrar on admission to a unit. They were approached for participation in the study after consent was received from their treating psychiatrists.

The CNC sample comprised 100 women recruited from the community. Women who were underweight (BMI < 18.5 kg/m^2^, *n* = 4) or overweight (BMI ≥ 25 kg/m^2^, *n* = 44) [48] were excluded. Normal-weight women who reported current or past eating disorder diagnoses and/or the practice of pathological weight control strategies such as laxative use were excluded. No screening for mental disorders was undertaken; however, those who exceeded the recommended normal cut-off point of 14 for DFT [29] or 20 on the depression and anxiety scales of the Depression, Anxiety and Stress Scale [49] were excluded.

### Measures

*Demographic and AN-related information.* Descriptive self-report data included age, education and employment status. Self-reported duration of the disorder, lowest and highest weights, feared and ideal weights (all at adult height), history of previous eating disorders, and exercise and binge/purge behaviours were recorded. They helped to describe the AN groups and to exclude possibly unsuitable community respondents from the CNC group. BMI was calculated for patients using clinician-recorded height and weight at the time of recruitment; for CNC participants, self-reported height and weight were used [50].

*Metacognitions Questionnaire—Brief Version* (MCQ-30) [51]: The MCQ-30 consists of 30 self-report items measuring five six-item subscales: *positive beliefs about worry*, *negative beliefs about worry*, *beliefs about the need to control thoughts*, *cognitive confidence*, and *cognitive self-consciousness*. Items are rated on a four-point Likert scale rated 1 (*Do not agree*), 2 (*Agree slightly*), 3 (*Agree moderately*) and 4 (*Agree very much*). Higher subscale and total scores indicate more dysfunctional metacognitive styles.

The MCQ-30 has been shown to retain the factor structure of the original MCQ and has construct validity [24]. Cronbach’s alphas for the subscales ranged from .72 to .93 and test-retest reliabilities from .59 to .87 with strong internal consistency in anorexia nervosa (λ = .98). For the present study, Cronbach’s alpha ranged from .90 to .95 for the five subscales in each group, indicating a high level of internal consistency.

*Eating Disorders Inventory-3* (EDI-3) [29]: The EDI-3 is a 91-item self-report inventory with 11 subscales, measuring attitudes, symptoms, and behaviours associated with eating pathology. Statements are anchored on a six-point Likert scale, some of which are reverse scored. Scores for positively scored items are weighted as follows: 4 (*Always*), 3 (*Usually*), 2 (*Often*), 1 (*Sometimes*), 0 (*Rarely*), 0 (*Never*), while reverse-scored items are weighted in the opposite direction. Higher scores represent higher levels of eating disorder pathology. The subscales have good convergent and discriminant validity. Test-retest reliability in women with eating disorders is high [52]. DFT is the only subscale reported in this study. It has seven self-report items measuring excessive preoccupation with achieving or maintaining thinness, and has good test-retest reliability (*r* = .92) and internal consistency, with Cronbach’s alpha ranging from .80 to .91 in clinical samples. Cronbach’s alpha in the present study was .86 in the patient sample and .90 in the non-clinical sample.

The questionnaire pack, which took about 40 min to complete, contained the above measures, questionnaires not included in the present study, the information brochure and consent form approved by the research and ethics committees, and information for removal of data at a later date.

### Procedure

Ethics approval was obtained from the research and ethics committees at The Royal Melbourne Hospital, The Melbourne Clinic and The University of Melbourne. All patients underwent a formal diagnostic interview with a consultant before admission to an eating disorders unit. Following the interviewer’s consent, individual patients were approached by a research student. The patients were informed verbally and in writing of the research objectives and the voluntary and confidential nature of participation. They were assured that participation would not affect their treatment. Questions were answered. After giving written consent, each respondent completed a questionnaire pack and returned it, sealed, to the nurses’ station or to the researchers. The completed questionnaires were stored separately from consent forms.

For the non-clinical sample, data were collected through the distribution of the questionnaire packs to first and second-degree acquaintances of student researchers using a snowballing technique. The method was chosen to better represent the age range in the patient group than obtained from a university student volunteer pool and to avoid related biases. The packs also included instructions for questionnaire completion and return, information enabling participants to judge whether they were underweight, and contact phone numbers for eating-related concerns. Consent was implied by return of the questionnaires in pre-paid, addressed envelopes. For further preservation of anonymity, each researcher entered data from another researcher’s assigned questionnaires rather than their own. Identification numbers on the return envelope and questionnaire set differentiated participants.

### Analyses

SPSS-22 was used for all analyses [53]. Preliminary testing indicated that for all scales skewness and kurtosis were within limits unlikely to significantly affect parameter estimates [54]. Considering the noted robustness of MANOVA to violations of homogeneity of variance and normality assumptions [54, 55], MANOVAs were used for group comparisons. Bonferroni corrections for the number of comparisons [56], gave *p* < 0.0028 for two-tailed testing of statistical significance. Effect sizes of differences between group measures were defined as partial η^2^ < .01 (small); <.06 (medium), and > .14 (large) [56]. Group sizes needed for power (*P*) = .8 were calculated *post hoc* from the standardised differences of the present study.

For combined testing of H1, H2 and H3, DFT and the five MCQ-30 factors were compared across AN-t and AN-at, then the two AN groups with CNC. The 95 % confidence intervals (*CI*s) for the means of each measure were inspected for their separation across groups, indicating significant differences. For DFT in AN-t and AN-at, the 90 % *CI* of the means difference was inspected to see if it could contain a *clinically* significant difference [57], taken as >5. For the exploratory H4, duration of AN and weight histories were compared in AN-t and AN-at by MANOVA without Bonferroni correction (*p* < 0.05). Exploratory zero order correlations were calculated. Non-parametric Mann–Whitney U tests checked for whether violation of assumptions in MANOVA led to different results.

For H5, to test for the impact of metacognitive factors on drive for thinness, separate regression analyses for each group were performed. Concurrent regression analyses have predictive limitations compared with longitudinal studies but are robust [58, 59]. With DFT as dependent variable, the independent variables (the five MCQ-30 scales) had stepwise entry with *p* < .05 and *p* > 0.1 for entry or removal of a variable, respectively. Power was calculated a priori [60] for the smallest group (*n* = 44) from *R*^2^ = .25, v = 5, *p* < .05 and effect size (*f* ^2^ = *R*^2^/1-*R*^2^) = .333, giving *P* = 0.8.

Nominal demographic data for group description were compared using the Chi-square statistic and *p* < .05, without correction for the number of tests performed.

## Results

### Descriptive statistics

The patient groups (AN-t *n* = 75; AN-at *n* = 44) and the CNC group (*n* = 100), were similar in age (*M* = 24.5 [*SD* = 6.9] years; *M* = 25.4 [*SD* = 6.9] years; *M* = 25.7 [*SD* = 7.1] years, respectively), *F*(2,217) = 1.17, *p* > .05, but differed in BMI (*M* = 16.0 [*SD* = 1.8] kg/m^2^; *M* = 21.0 [*SD* = 1.9] kg/m^2^; *M* = 21.7 [*SD* = 1.9] kg/m^2^; respectively), *F*(2,217) = 188.1, *p* < .001. The CNC group had attained a higher level of education (χ^*2*^(5,*N* = 214) = 39.02, *p* < .05) and were more likely to be currently working (χ^*2*^(2, *N* = 217) = 22.45, *p* < .05) while AN-t and AN-at did not differ significantly for either (*p* > .05).

### Comparison of AN-t, AN-at and CNC

Using MANOVA, group differences between AN-t and AN-at were not significant *(F*(1,117) = 1.28, *p* > .05, partial ƞ^2^ < .01) (Table 1). For each measure, the 95 % *CI*s overlapped; for DFT the 90 % *CI* around the mean difference (−4.488 to 0.400) was 4.9 units, not considered clinically significant. Observed power was *P* < .3. For significant differences at *p* < .05, and *P* = .8, and using the study’s standardised differences between mean measures in AN-t and AN-at, sample sizes would range between *n* > 350 to *n* > 1100. Hypotheses 1 and 2 could not be accepted; the groups were equivalent.

**Table 1:** Comparison of AN patients, split into typical (BMI < 18.5 kg/m^2^) and atypical groups (BMI ≥ 18.5 kg/m^2^) and a community-based non-clinical group, showing means and standard deviations (M(SD)), 95 % confidence intervals, individual F-test results from MANOVA and partial effect sizes of differences between groups

	Typical AN		Atypical AN		CNC			
	(*n* = 75)		(*n* = 44)		(*n* = 100)			
Measures	M(SD)	95 % CI	M(SD)	95 % CI	M(SD)	95 % CI	F(2,216)	Partial η^2^
DFT	18.5(7.6)	16.9 – 20.1	21.0^a^(5.8)	18.9 – 23.1	4.9***(4.4)	3.7 – 6.1	164.0	.603
MCQ-CC	12.7(5.0)	11.6 – 13.9	12.3(5.2)	10.8 – 13.8	9.3***(3.5)	8.4 – 10.1	15.1	.123
MCQ-PBW	10.9(4.8)	9.7 – 12.1	12.0(5.9)	10.4 – 13.6	9.1^b^***(3.8)	8.1 –10.0	7.2	.062
MCQ-CSC	17.5(4.2)	16.5 – 18.5	16.8(4.6)	15.5 – 18.1	13.3***(5.0)	12.4 – 14.2	19.6	.154
MCQ-NBW	17.8(4.7)	16.7 – 18.9	17.8(5.0)	16.4 – 18.3	8.7***(3.7)	7.9 – 9.6	119.6	.526
MCQ-NCT	15.2(5.0)	14.0 – 16.3	16.0(5.1)	14.5 – 17.5	8.7***(3.1)	7.9 – 9.6	68.3	.387

*Note.* DFT: Drive for Thinness subscale of the Eating Disorders Inventory-3 [29]; MCQ-30: Metacognitions Questionnaire—Brief Version [51] with MCQ-CC: Cognitive Confidence, MCQ-PBW: Positive Beliefs About Worry, MCQ-CSC: Cognitive Self-Consciousness, MCQ-NBW: Negative Beliefs about Worry and MCQ-NCT: Need to Control Thoughts

Partial η^2^ < .01 small, <.06 medium and < .14 large [57]

*p < .05, **p < .01, ***p < .001 comparing the community group with both AN groups

^a^ *p* < .05 comparing typical with atypical AN. ^b^ *p* < .01 comparing the community group with typical AN

Differences between the AN and CNC groups (Table 1) were highly significant with large effect sizes (*F*(6,212) = 76.9, *p* < .001, partial ƞ^2^ = .658) and *P* = 1. The 95 % *CI*s for each measure in AN-t and AN-at did not overlap those of the CNC group, except for *positive beliefs about worry* with AN-t. The Mann–Whitney U tests confirmed the findings, except for finding a greater significance for *positive beliefs about worry* between AN-t and CNC. H2 was supported, the AN and CNC groups were not equivalent.

### Exploratory comparisons of AN-related variables

Duration of the disorder was similar in AN-t and AN-at but BMI-related measures were significantly different (Table 2). For all but eight AN-at patients, the reported lowest BMI was less than 18.5 kg/m^2^ indicating that 36 (82 %) had met the study’s BMI criterion for AN-t in the past and 75 % reported less than 17.5 kg/m^2^. Mean ideal BMI was less than current BMI in both groups, with its distance from the current BMI being similar (*p* > .05). Regarding measures for AN typology, binge/purge behaviour (59 % of AN-t and 69 % of AN-at) was similar (χ^2^(1, *N* = 119) = .095, *NS*), as was exercise for controlling weight (80 % of AN-t and 84 % of AN-at) (χ^2^(1, *N* = 119) = .091, *NS*). In AN-t, duration was related to *need to control thoughts* (*p* < .003).

**Table 2:** Comparison of anorexia-related measures in typical AN (BMI < 18.5 kg/m^2^) and atypical AN (BMI ≥ 18.5 kg/m^2^) by ANOVA

	Typical AN		Atypical AN			
Measures	n	Mean (SD)	n	Mean (SD)	F	Partial η^2^
Duration of disorder (yr)	62	8.5 (7.9)	36	10.0 (6.6)	0.95	NS
Lowest BMI (kg/m^2^)	67	14.0 (1.7)	43	16.5 (2.6)***	38.1	.262
Highest BMI (kg/m^2^)	62	22.9 (6.5)	39	25.7 (4.6)*	5.63	.054
Feared BMI (kg/m^2^)	67	19.0 (2.7)	43	21.4 (2.8)***	20.3	.158
Ideal BMI (kg/m^2^)	66	17.3 (1.9)	44	19.3 (2.2)***	23.2	.177

*Note*. Partial η^2^ < .01 small, <.06 medium and < .14 large [57]

*p < .05, **p < .01, ***p < .001 comparing typical with atypical AN

### Preliminary correlations

Inspecting correlation coefficients in each group with corrected *p* < .0028, BMI, a possible covariate in the regression analyses, was unrelated to any measure. Correlations between the MCQ-30 factors were significant in each group for *negative beliefs about worry, need to control thoughts* and *cognitive self-consciousness* (*p* < .001), indicating non-orthogonality.

### Regression analysis

In both AN groups, the stepwise regression models concurrently predicting DFT were significant (Table 3). In AN-t, *positive beliefs about worry* and *need to control thoughts* were significant predictors (21 % of shared variance). In AN-at, *negative beliefs about worry* and, inversely, *cognitive self-consciousness* accounted for 29 % of shared variance. In the CNC group, *cognitive self-consciousness* accounted directly for a small 7 % of the variance. H5 was partially supported for each group.

**Table 3:** Summary of stepwise regression analysis predicting Drive for Thinness in female patients with typical anorexia nervosa (*n* = 75), atypical anorexia nervosa (*n* = 44) and a non-eating disordered community sample (*n* = 100)

	Model 1			Model 2					
Variable	B	SE B	β	B	SE B	β	R^2^	F change	df
Typical Anorexia Nervosa (*n* = 75)									
MCQ-PBW	.566	.174	.358	.501	.168	.315**	.127	10.58**	1,73
MCQ-NCT				.452	.162	.295**	.212	7.82**	2,72
Atypical Anorexia Nervosa (*n* = 44)									
MCQ-NBW	.516	.160	.446	.778	.190	.672**	.199	10.4**	1,42
MCQ-CSC				-.421	.182	-.378	.291	5.3*	2,41
Community-based non-clinical (*n* = 100)									
MCQ-CSC	.286	.106	.262				.069	7.22**	1,98

*Note.* Drive for Thinness: Drive for Thinness subscale of The Eating Disorders Inventory-3 [29]; MCQ-30: Metacognitions Questionnaire—Brief Version [51] with MCQ-PBW: Positive Beliefs About Worry, MCQ-NCT: Need to Control Thoughts, and MCQ-CSC: Cognitive Self-Consciousness

*p < .05, **p < .01

## Discussion

This study investigated in women metacognition and drive for thinness, a core aspect of the psychopathology of anorexia nervosa. Drive for thinness was equally strong in typical and atypical anorexia nervosa. Previous findings that four of the five metacognitive factors in patients were more dysfunctional compared to controls [32–34] were confirmed. The fifth, *positive beliefs about worry,* was also significantly greater than in the non-clinical group. The dysfunction was equally apparent in typical and atypical anorexia nervosa. Drive for thinness was concurrently predicted by *positive beliefs about worry* in the typical anorexia nervosa group and by *negative beliefs about worry* in the atypical group. The finding that both positive and negative cognitions were related to drive for thinness, also seen across groups in the correlational analyses, supported the importance of cognitive processes for anorexia nervosa [22]. *Need to control thoughts* was the second contributory factor in the typical group while an inverse relationship was revealed between drive for thinness and *cognitive self-consciousness*. In the non-clinical group, drive for thinness was predicted by *cognitive self-consciousness*, a factor previously related to obsessive-compulsive symptoms [43].

Compared with the non-clinical group, women with anorexia nervosa had less confidence in their cognitive functioning, were more aware of their worrying thoughts, experienced worry as more dangerous and uncontrollable, yet saw greater benefits in worrying. They also believed more strongly in the need to control their thoughts to avoid negative consequences. The findings corroborate and broaden previous descriptions of poor sense of control in anorexia nervosa [38], demonstrating that the perceived lack of, and struggle for, control extends to the higher levels of cognition. They also showed that, on the primary measures of the study, the groups were virtually indistinguishable; to find significance at *P* = .8 would require sample sizes of over 350 for drive for thinness and over 1100 for the MCQ-30 factors, clearly ruling out the clinical significance of differences between the groups. The findings support the clinical and patient observation that weight is not an accurate indicator of the severity of the disorder, or of recovery. It also suggests that being of normal weight does not equate with normal metacognition in anorexia nervosa.

The exploratory hypotheses added a greater understanding of the atypical group through their self-reported weight history. The weight-related measures, highest, lowest, feared and ideal weights converted to BMI, were significantly greater in the atypical group, yet all but a handful had fulfilled the study’s weight criterion for anorexia nervosa in the past. Despite having restored weight to within the normal range they still exhibited clinically significant symptoms requiring intensive treatment. Furthermore, ideal weights reported by both groups were below their present weights. That, taken together with their high drive for thinness, confirms their continued need for treatment. Both groups had similar duration of their eating disorders and proportions of binge/purge behaviour and exercising for weight control. Although the results are inconclusive, it is possible that we are observing a trans-diagnostic phenomenon [23, 37] or the transitioning of the majority of atypical patients through a weight-related trajectory across the two diagnostic groups.

Drive for thinness, by being concurrently predicted by *positive* or *negative beliefs about worry*, in the patient groups, demonstrated the embrace of their particular styles of worrying to support their striving for weight loss. In the typical group, it was also concurrently predicted by the metacognitive factor in which control is central, the *need to control thoughts.* It suggests that the perception of poor control at both the cognitive and process levels helps drive the determination to be thinner [38]. An unexpected finding in the atypical group was the inverse relationship between drive for thinness and *cognitive self-consciousness*, revealed after the entry of *negative beliefs about worry* into the regression equation. The zero order correlation had been positive. C*ognitive self-consciousness* in psychopathology, with its heightened awareness of cognitive processes and their rigid monitoring and narrow focus on anorexia-related cognitions [40–42] was hypothesised to predict concurrently, drive for thinness in anorexia nervosa, as was apparent in the non-clinical group. The opposite was found. The lower the *cognitive self-consciousness*, the stronger the drive for thinness. The role of this metacognitive factor requires further investigation.

The broad findings confirm that anorexia nervosa could be added to the disorders associated with the CAS, and fits well with the its trans-diagnostic nature, both across the separate diagnostic categories of the eating disorders [17, 32] and other mental disorders assimilated into the CAS model.

### Clinical implications

The findings suggest that distress and obsession around weight may be driven, at least in part, by dysfunctional metacognitive beliefs, for which current practices of cognitive behavioural therapy and pharmacotherapy are inadequate [1, 10]. These results support the continued testing of process-based therapies [16, 17] to ameliorate drive for thinness. Given that a relatively small amount of variance in drive for thinness was explained by metacognitive factors, the clinical significance of the findings remains uncertain; however, the results suggest that further investigation would be productive. In addition, when offering treatment to patients with atypical anorexia nervosa, the severity of psychological dysfunction should be taken into account.

An intriguing finding was the similarity in frequencies of behaviours related to binge/purge type (about two-thirds of patients) and exercising (over 80 %) in the patient groups. While it may be partially a consequence of the patient selection process, the finding still requires further study to benefit the design of therapeutic programs.

The contribution to drive for thinness in the non-clinical community sample of *cognitive self-consciousness*, already noted as related to potential dysfunction [43] could be considered when designing community health prevention programs.

### Implications for theory

The findings of the study support the S-REF model [19, 20, 40] as being relevant to anorexia nervosa, with the inclusion of cognitive *processes* rather than cognitive *content*. They contribute to our understanding of the preoccupation with becoming thinner, characteristic of sufferers of anorexia nervosa. The findings add to the evidence for the role of dysfunctional metacognitions in psychopathology by not only confirming their relevance to anorexia nervosa [22, 25–27] but also demonstrating their relationship to its psychopathology through drive for thinness. It would appear that dysfunctional metacognition serves to maintain anorexia nervosa. Theories suggesting that *positive* and *negative beliefs about worry* generate heightened worry engagement, concentration on fears, and avoidance behaviours in psychopathology [32–36] appear to include sufferers of anorexia nervosa.

### Implications for research

A pressing need for future research is the development of relevant criteria to distinguish typical and atypical anorexia nervosa, to enhance inter-study comparisons and therapeutic trials [43]. The criterion value adopted for the present study was based on defining atypical anorexia nervosa to have a minimal normal BMI ≥ 18.5 kg/m^2^, equivalent to that of the normal population. As a consequence, a maximum BMI for defining typical anorexia nervosa was also established, creating a category between BMI ≥ 17.0 kg/m^2^ to BMI < 18.5 kg/m^2^ defined as mildly severe. It is apparent that a consequence of determining BMI-based categories of severity [43] is the need for research to test their meaning and relevance for theory, therapy, and the ramifications for considering later diagnostic changes to the eating disorders.

Other classificatory uncertainties [45, 46] for further study in adult typical and atypical anorexia nervosa include another possible criterion, rate of recent weight loss, important because rapid weight loss contributes to medical instability [9], bringing the patient to the notice of health professionals [43]. In future studies, rate of pre-admission weight loss could be recorded.

In order to accurately apply the S-REF model to inform future treatment, it is important to clarify whether anorexia nervosa involves a metacognitive profile distinct from those of other psychological disorders. The non-orthogonal nature of the MCQ-30 scales limits researchers’ confidence in isolating metacognitive factors in anorexia nervosa. Sample sizes large enough to conduct structural equation modelling and factor analysis are essential [59]. Furthermore, duplication of results in considerably larger and equal sample sizes is essential to improve reliability when comparing groups and interpreting results.

### Limitations of the study

The findings of this study must be interpreted in the context of limitations, some of which have already been discussed above.

A major limitation is small sample size, which constrained statistical testing in the groups with anorexia nervosa. While power for regression was adequate for the smallest group (*P* = .8), the number of variables awaiting entry at each step was large [53], and could have led to foreclosure prior to full testing of the model, thus excluding entry of other potentially significant variables and producing a conservative model. Low numbers also prevented the use of hierarchical regression for model testing in randomised half-samples from each group. Conversely, markedly large sample sizes for MANOVA would be required to detect statistically significant differences in drive for thinness and the metacognitive factors between typical and atypical anorexia nervosa for the measures of this study. Clinical significance would then have little meaning.

Another limitation of the regression analysis is the concurrent sampling of all measures [59], which prevents inferences of causality between metacognitive factors and drive for thinness. A longitudinal study would address this limitation and also answer questions about movement of patients across diagnostic groups.

Additionally, despite being informed that participation was anonymous and unrelated to treatment, it remains possible that some patients misreported responses to the questionnaires in an attempt to receive greater attention from staff or to achieve early discharge. In the non-clinical sample, the calculation of BMI was based on self-reported weight and height which, despite evidence for its reliability [50], may have reduced accuracy. The reliance on self-report and questionnaire-based exclusion criteria to eliminate respondents who may have suffered previously from an eating disorder may be misplaced, suggesting the inclusion of the more stringent but expensive assessment interview for non-clinical participants.

Conclusions drawn from the study are limited by the exclusion of men from analysis. Too few had been admitted for treatment for statistical analysis.

## Conclusions

The findings of the study support emerging knowledge about metacognitive dysfunction in the maintenance of anorexia nervosa, and expand the empirical evidence base regarding the underlying cognitive processes of the disorder. Ultimately, the study suggests that continued exploration of process-based therapies for anorexia nervosa is worthwhile. All facets of metacognition were found to be more dysfunctional in the patients than in the non-clinical sample. In both typical and atypical anorexia nervosa, they were similar, yet had different, independent associations with drive for thinness. Drive for thinness itself was similarly severe in the two groups. Differentiation of the patient sample into typical and atypical anorexia was warranted by the findings but the process of differentiation is open to further study. That a number of patients with atypical anorexia nervosa had previously fulfilled the weight criterion for anorexia nervosa suggests caution and consideration of transitional staging of eating disorders. The inclusion of a non-clinical sample reporting no previous eating disorders and showing a link, albeit weak, between metacognition and drive for thinness, indicated background social effects on metacognition and suggests an area for health promotion activities.

## Abbreviations

AN: Anorexia nervosa
AN-t: Typical anorexia nervosa
AN-at: Atypical anorexia nervosa
BMI: Body mass index
CAS: Cognitive attentional syndrome
CNC: Community-based non-clinical participants
DFT: Drive for thinness subscale of the Eating Disorders Inventory-3
DSM-5: Diagnostic and Statistical Manual of Mental disorders, Fifth edition
EDI-3: Eating Disorders Inventory-3
MANOVA: Multivariate analyses of variance
MCQ: Metacognitions questionnaire
MCQ-30: Metacognitions questionnaire—brief version
S-REF: Self-regulatory executive function
